# Co-occurrence of acute leukemia and type 1 diabetes among children: a caseseries

**DOI:** 10.1186/s13256-026-06077-w

**Published:** 2026-05-29

**Authors:** Henri Rosti, Julia Ventelä, Olli Lohi, Atte Nikkilä

**Affiliations:** 1 TamCAM - Tampere Center for Child, Adolescent and Maternal Health Research, Tampere University, Tampere, Finland; 2 Tays Cancer Centre and Department of Pediatrics, Tampere University Hospital, Tampere, Finland

**Keywords:** Acute leukemia, Type 1 diabetes mellitus, Pediatrics

## Abstract

**Background:**

Leukemia is the most common malignancy in children, while type 1 diabetes mellitus (T1DM) is one of the most prevalent autoimmune diseases among children. The etiology of both conditions is still largely unexplained, yet emerging evidence suggests certain similarities in environmental exposures and genetic predispositions. In this case-series, we sought a new potential factor behind the co-occurrence of leukemia and T1DM by thoroughly reviewing the medical records of patients diagnosed with both diseases.

**Methods:**

We conducted a retrospective case series at Tampere University Hospital, Finland, by identifying pediatric patients diagnosed with both acute leukemia and T1DM from 1990 to 2023 using (International Classification of Diseases-10th revision) ICD-10 codes. Clinical and laboratory data, including leukemia subtype, age at diagnosis, treatments, comorbidities, and medical history, were collected, reviewed and analyzed descriptively. Study protocol was locally approved, and patient health data privacy was respected.

**Results:**

Among 12 initially identified cases, seven met the inclusion criteria. Five cases were diagnosed with B-cell acute lymphoblastic leukemia (B-ALL), one with T-cell ALL (T-ALL), and one with acute myeloid leukemia. The mean age at leukemia diagnosis was 8.7 years (range from 0.7 to 15.3), while the mean age at T1DM diagnosis was 11.8 years (range from 7.5 to 17.8). In five cases, leukemia preceded T1DM, with two cases linked to asparaginase-induced pancreatitis. In two cases, T1DM developed before leukemia. Additional comorbidities included Down syndrome, Sotos syndrome, celiac disease, epilepsy, and Sweet’s syndrome.

**Conclusion:**

This case-series presents findings, which align with previous observations (e.g., Down syndrome, pancreatitis), though a less often reported finding, Sotos syndrome, was also observed.

## Background

Leukemia is the most common malignancy among children under 15 years of age with acute lymphoblastic leukemia (ALL) being the most common type (85%) followed by acute myeloid leukemia (AML, 10–15%). Leukemia is also the fourth most common cause of death among children [1].

Incidence of type 1 diabetes mellitus (T1DM) in Finland is one of the highest globally, and T1DM is one of the most common autoimmune diseases among children [1]. During the years 1987 to 1991 the incidence rate varied from 4 to 245 per 100 000 children between municipalities in Finland [2]. In 2025, the national incidence of T1DM in Finland was 64.2 per 100,000 children under the age of 15 while the global incidence in 2025 was 8.2 per 100,000 in the same age group [3]. These figures indicate that Finland has one of the highest incidence rates of T1DM in the world, with a markedly faster increase over time compared to global trends, suggesting the influence of unique environmental or lifestyle factors.

The exact etiologies of both acute leukemia and T1DM remain largely unclear. Certain treatments for ALL, such as asparaginase, may induce diabetes through severe pancreatitis, while corticosteroid therapy can cause transient hyperglycemia requiring insulin management [4]. Patients with Down syndrome are predisposed to both acute leukemia and T1DM, further highlighting potential shared mechanisms [5]. Nevertheless, even after accounting for these factors, emerging evidence indicates that T1DM remains significantly associated with acute leukemia, although the underlying cause of this relationship is still unknown [1, 6–8]. Previous studies have suggested that the two diseases may share common infectious triggers, or that metabolic disturbances, linked to diabetes, could contribute to leukemogenesis [6, 9]. Additionally, genetic factors such as alterations in *PTPN22* and *IKZF1* genes have been implicated in both conditions, suggesting the possibility of certain shared genetic susceptibility between T1DM and acute leukemia [10, 11].

To better understand the connection between T1DM and acute leukemia, we carefully reviewed all available medical charts of patients diagnosed with both diseases in our tertiary children’s hospital. Our purpose was to explore if patient medical records would hold information that could help in explaining the co-occurrence.

## Methods

We sought to present a comprehensive description of the clinical progression in this case series and to identify possible contributing factors to the observed association by thoroughly reviewing each patient’s medical records.

This case-series was conducted at the children’s and adolescents’ hospital in Tampere University Hospital, Tampere, Finland. The cases with both diseases from 1990 to 2023 were identified by the hospital’s IT department using ICD-10 diagnosis codes, and the subjects were required to be under 18 years old at the timepoints both diagnoses were placed.

After the hospital’s IT department had identified the cases, we checked whether they met the given criteria. We reviewed the following information to confirm the diagnosis of diabetes: in symptomatic patients (with thirst, weight loss, and polyuria), diagnostic criteria included a random plasma glucose level > 11 mmol/L, fasting plasma glucose > 6.9 mmol/L, or HbA1c > 48 mmol/mol. In asymptomatic individuals, a second confirmatory measurement was required. The final diagnosis was verified by a pediatric endocrinologist, and initial assessments included C-peptide measurement and screening for diabetes-associated autoantibodies such as islet cell antibodies and insulin antibodies [12]. Additionally, the accuracy of the diagnosis was confirmed by The Social Insurance Institution of Finland (Kela) based on specific medical certificate.

The following clinical and laboratory data were collected and tabulated from the medical charts: age at both diagnoses, genetics and phenotypic subtype of leukemia, all treatments for both diseases, and every other comorbidity the subjects had. Data on growth, development and social history were also collected.

The data were analyzed descriptively, and the study was conducted following ethical standards of our hospital district, with patient health data privacy maintained and person-identifying information excluded at the analysis stage. The study protocol was approved by the Chief medical officer of the hospital services division, Wellbeing Services county of Pirkanmaa.

## Results

A query to the hospital`s medical registry initially identified 12 individuals with both acute leukemia and T1DM. However, five cases were excluded after further inspection: one subject had type 2 diabetes mellitus, one had no data available due to moving to another area and one subject had only transient hyperglycemia without a confirmed diagnosis of T1DM. Moreover, two cases did not have leukemia.

**Fig. 1:**
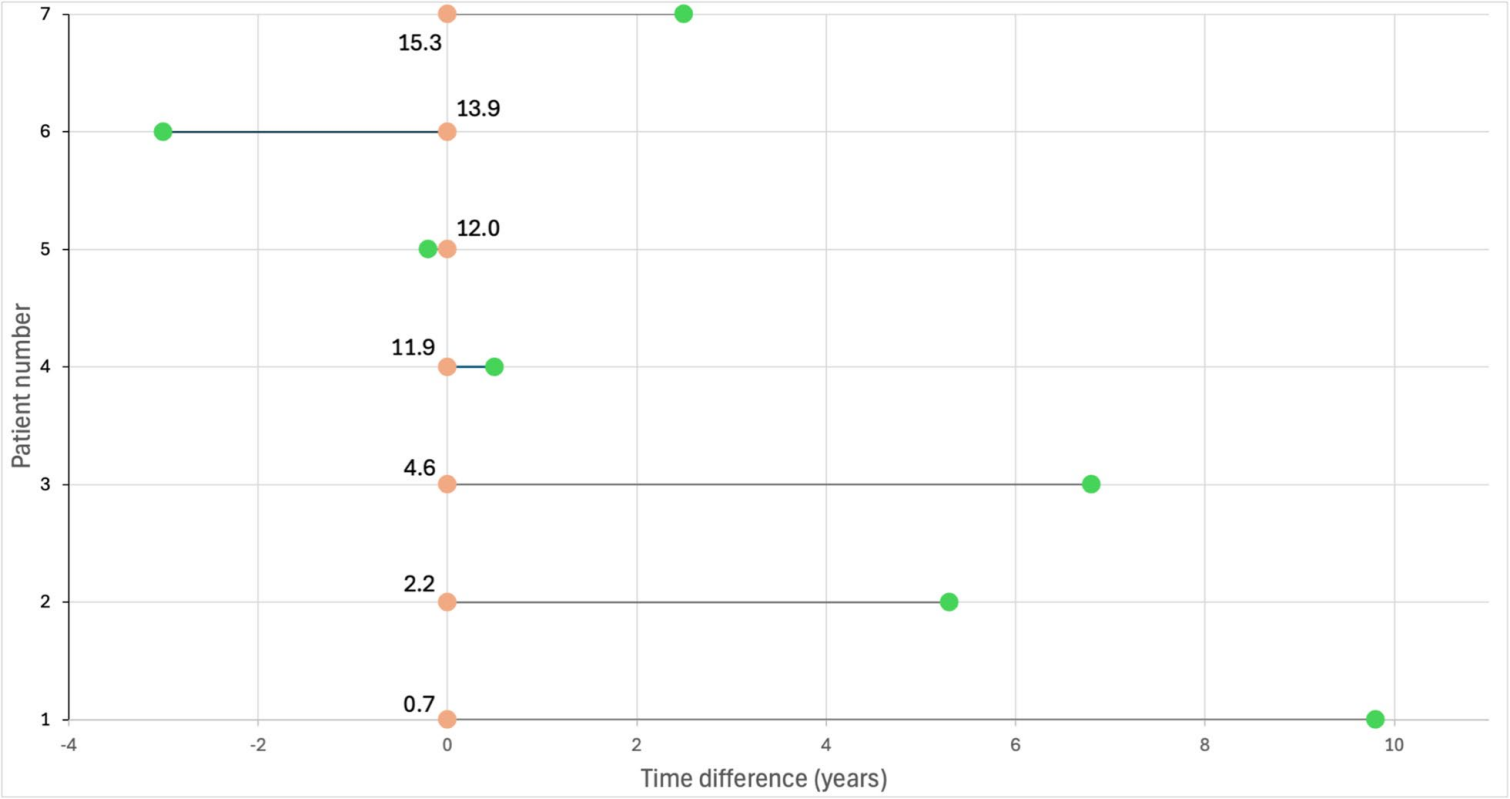
Time difference between leukemia and type 1 diabetes mellitus (T1DM) diagnoses. Orange dots mark acute leukemia diagnosis and the numbers next to them represent patients’ age at the time of diagnosis. The green dots denote T1DM, and the grey line represents the time difference between acute leukemia and T1DM diagnoses

In total, seven cases with both diseases were identified (Fig. 1). Five out of seven cases were diagnosed with B-ALL: one with *Pax5alt* subtype, another with *TCF3-PBX1* subtype, while the rest did not have subtype information available. In addition, one case of T-ALL and one AML (M7) were identified. The mean age at leukemia diagnosis was 8.7 years (range 0.7–15.3), while the mean age at diabetes diagnosis was 11.8 years (range 7.5–17.8).

Out of six cases with ALL, five had a leukemia diagnosis preceding the onset of T1DM, with the time difference between diagnoses ranging from + 0.5 to + 9.8 years. Two cases developed T1DM following acute pancreatitis caused by asparaginase treatment, whereas two cases were diagnosed with T1DM 5.3 (B-ALL) or 6.8 (B-ALL) years after leukemia diagnosis and had no obvious connection to leukemia treatment. Two cases with either a T-ALL or B-ALL had T1DM diagnosis prior to leukemia diagnosis (−0.2 and −3.0 years, respectively). The only case with AML had T1DM diagnosed 9.8 years after leukemia diagnosis.

Additionally, the subjects suffered from a few other medical conditions such as Down syndrome, celiac disease, Sotos syndrome, epilepsy, migraine, polycystic ovary syndrome and Sweet’s syndrome (Table 1).

**Table 1:**
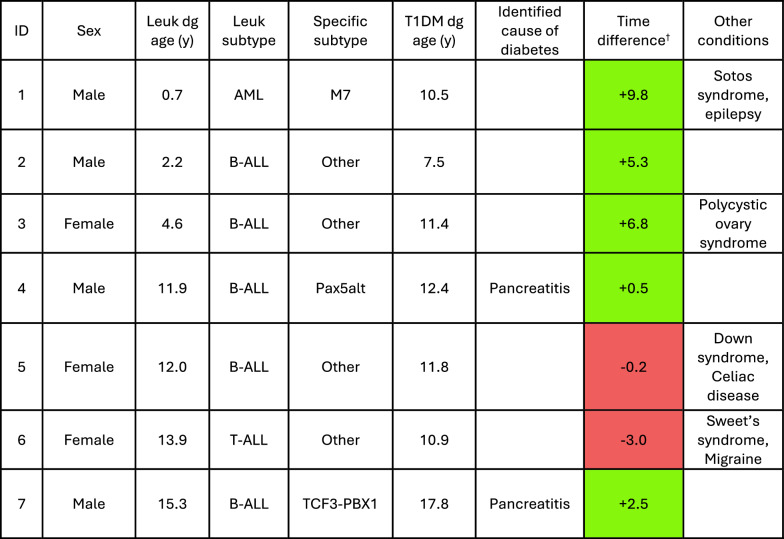
Patients’ clinical characteristics

^†^ *Age at type 1 diabetes mellitus (T1DM) diagnosi*s was subtracted from *Age at leukemia diagnosis*. Thus, “ + ”-sign represents that type 1 diabetes was diagnosed prior leukemia and “-”-sign represents that leukemia was diagnosed prior type 1 diabetes

## Discussion

The co-occurrence of T1DM and childhood acute leukemia has been repeatedly documented. In this case series, we aimed to provide a detailed account of the clinical sequence of events and to reveal potential factors behind the association by going carefully through individual patient’s medical records. The number of cases with co-occurrence of both diseases was small in our hospital, and the study was not designed to determine whether the diseases occur together more often than expected. Nevertheless, we were able to identify known risk factors (pancreatitis and Down syndrome) as well as a previously unrecognized factor (Sotos syndrome). Notably, two cases were diagnosed with T1DM before leukemia, and two additional cases received a T1DM diagnosis years after completing leukemia treatment, further corroborating association form previous epidemiological research [6, 8].

For two subjects, T1DM was thought to be caused by pancreatitis, which was induced by asparaginase, leading to pancreatic tissue destruction and permanent insulin dependency. Of note, one of the patients who had T1DM prior leukemia, had also Down syndrome, a known risk factor for both conditions [5].

Down syndrome is a well-known risk factor for both leukemia and diabetes, whereas Sotos syndrome’s influence on the co-occurrence is unclear. Sotos syndrome is known to predispose to leukemia but its association with T1DM is still not clear, even though it has parallels to glucose metabolism [5, 13].

Additionally, Sweet syndrome was identified in an AML patient. Sweet syndrome is a rare inflammatory condition associated with malignancies but may not directly contribute to leukemia nor diabetes pathogenesis [14].

Our study highlights the limitations of hospital registry queries. Our initial hospital registry search identified 12 patients, but five of them did not meet the study inclusion criteria. This underscores the shortcomings of hospital registries and encourages careful review of the query results manually, if possible, and the importance of further review in registry-based research when relying solely on diagnosis codes.

It remains largely unclear how diabetes may promote malignant growth; however, metabolic disturbances and chronic hyperglycemia have been suggested to contribute to tumorigenesis [6]. It has also been observed that both ALL and AML show a higher incidence rate when leukemia develops after the onset of T1DM, indicating a possible influence of insulin therapy or shared environmental triggers [9]. According to the hygiene hypothesis, limited exposure to pathogens during early childhood may increase the risk of autoimmune diseases later in life [15]. Similarly, reduced exposure to infectious agents during infancy has been proposed to promote leukemogenesis [16]. However, although leukemia and T1DM share certain genetic susceptibilities and environmental factors, the extent to which these common background features contribute to their co-occurrence remains uncertain [10, 11].

However, overall evidence remains limited, as most prior studies have focused on adult populations or combined T1DM and T2DM cases. To date, only a few studies and case series have specifically examined association of pediatric T1DM and ALL, indicating that while a possible association exists, the underlying mechanisms and causal pathways require further investigation.

In summary, with this case series, we identified known risk factors for leukemia and T1DM, including Down syndrome and asparaginase-induced pancreatitis. Yet we observed a less frequently occurring factor, Sotos syndrome in one patient with AML.

## Conclusion

These findings are generally consistent with existing literature on established risk factors, such as Down syndrome and treatment-related pancreatitis, while also noting one less recognized factor, Sotos syndrome. The temporal variation between leukemia and T1DM onset emphasizes that their co-occurrence is not explained by leukemia treatment side-effects alone. Our findings highlight the need for further research into possible shared genetic predispositions, syndromic associations, or immunological mechanisms.

## Data Availability

The dataset supporting the conclusions of this article are not available due to privacy and confidentiality restrictions.

## Abbreviations

T1DM: Type 1 diabetes mellitus
AML: Acute myeloid leukemia
ALL: Acute lymphoblastic leukemia
